# Role of perigastric arcade removal in serous epithelial ovarian cancers

**DOI:** 10.3332/ecancer.2023.1519

**Published:** 2023-03-13

**Authors:** Rohini Vinayak Kulkarni, Manoranjan Mohapatra, Bhagyalaxmi Nayak, Sagarika Samantaray, Janmejaya Mohapatra, Jita Parija, Sushil Kumar Giri

**Affiliations:** 1 Department of Gynaecological Oncology, Tata Memorial Centre, Mumbai 400012, India; 2 Department of Gynecological Oncology, Acharya Harihar Post Graduate Institute of Cancer, Cuttack 753007, Odisha, India; 3 Department of Pathology, Acharya Harihar Post Graduate Institute of Cancer, Cuttack 753007, Odisha, India; a https://orcid.org/0000-0002-4466-1703; b https://orcid.org/0000-0001-9436-4072; c https://orcid.org/0000-0002-6319-5365; d https://orcid.org/0000-0001-7589-2953; e https://orcid.org/0000-0001-5577-3850; f https://orcid.org/0000-0001-8397-3921; g https://orcid.org/0000-0001-8158-7605

**Keywords:** ovarian cancer, omentectomy, perigastric arcade, micrometastasis

## Abstract

**Introduction:**

Omentectomy is an essential part of cytoreductive surgery (CRS). However, removal of perigastric arcade (PGA) of the omentum is a controversial aspect of omentectomy in view of the fear of injury, vascular compromise and gastroparesis. Hence, we conducted a study to evaluate the necessity and effect of removal of PGA during omentectomy.

**Methods:**

The nature of the study was a prospective observational study. The study period was for 1 year between 1.3.2019 and 29.2.2020. Patients with stage III to IV serous epithelial ovarian cancers – chemo naive/post neoadjuvant chemotherapy, without macroscopic involvement of the PGA were included in the study. Patients were divided into two groups – those who had PGA removed (group 1) and those whose PGA was preserved (group 2). Pre, intra and postoperative factors between the two groups were compared using standard statistical methods.

**Results:**

Micrometastasis to PGA was present in 36.4% of the patients in group 1. The predictors for this involvement included gross involvement and microscopic involvement of the mobile part of the omentum (*p* < 0.001), pre surgery Meyer’s score (*p* < 0.05) and requirement of peritonectomy (*p* < 0.05) during the CRS implying that higher the peritoneal carcinomatosis, more are the chances of microscopic involvement of PGA. On comparing postoperative outcomes between the two groups, we noted a statistically significant difference in intra-operative time (*p* < 0.01), prolonged recovery time with increased intensive care unit and hospital stay (*p* < 0.001) in group 1, although all with small absolute difference. However, there was no significant difference in major post-operative complications or time taken to tolerate soft diet.

**Conclusion:**

Micrometastasis to PGA was noted in significant number of cases. Its removal is also a safe procedure with minimal morbidity and good postoperative outcomes especially in cases with significant peritoneal carcinomatosis. Hence, it should be considered, provided we are achieving a complete cytoreduction otherwise.

## Introduction

Ovarian cancer is not only the second most common gynaecological malignancy affecting women in India, but is also the most lethal as per the Indian Cancer Registry [1, 2]. Almost 75% of these are diagnosed in advanced stage (stage III–IV) [3] and one of the most important predictors of survival is residual disease after cytoreductive surgery (CRS) [4, 5]. Most apparent early-stage epithelial ovarian cancers (EOC) are upstaged due to occult involvement of omentum and lymph nodes in up to 30% of cases, of which 10%–30% involve the supracolic omentum [8, 9]. Despite omentum being an established sanctuary for EOC metastasis, the extent of its removal has not been addressed appropriately till date [7]. Removal of perigastric arcade (PGA) of the omentum is one of the controversial aspects of omentectomy in view of its proximity to the stomach and hence the fear of injury, vascular compromise and gastroparesis. Cordeiro Vidal *et al* [10] in their study found microscopic involvement of the PGA in advanced epithelial ovarian cancer (AEOC) in 62.5% of the cases. But, the implications of this procedure on intra and postoperative complications have not been addressed adequately [9, 10].

Hence, in an attempt to fill the existing lacunae in the recent evidence, we conducted the current study with the aim to assess the distribution of microscopic disease in the PGA (including the left and right gastro-epiploic vessels) in patients of AEOC without macroscopic involvement of the PGA and to compare the intra-operative and immediate post-operative complications and course of recovery between patients who had removal of PGA and those who had the arcade retained during omentectomy.

## Methods

Patients with stage III to IV serous EOC – chemo naive/post neoadjuvant chemotherapy (NACT), without macroscopic involvement of the PGA were included in the study.

Early-stage epithelial serous cancer, non-epithelial, non-serous ovarian cancer, recurrences of ovarian cancer and those with macroscopic involvement of the PGA were excluded.

The study was initiated after Institutional Ethics Committee approval which was obtained on 22.2.2019. The study period was for 1 year between 1.3.2019 and 29.2.2020. The nature of the study was a prospective observational study. The study was conducted in the Department of Gynaecological Oncology of the institute, within two units of the department. One unit had a protocol wherein during omentectomy, the PGA was spared, while the other unit protocol was to remove the PGA. We included patients who had removal of PGA during omentectomy into group 1 and those whose PGA was retained during omentectomy into group 2. Written informed consent was obtained from all study participants.

As a part of routine preoperative evaluation, contrast-enhanced computerised tomography (CECT) of thorax, abdomen and pelvis was performed in all of them to evaluate the operability using Mayer’s score. A score of less than 3 was considered operable. Patients with a score ≥ 3 underwent NACT. All patients underwent Cancer Antigen (CA) 125 pre operatively. The ones who underwent NACT were re-evaluated with CECT & CA125 preoperatively.

Xiphopubic midline skin incision was performed. The extent of peritoneal disease was assessed using Peritoneal Cancer Index (PCI) [11].

Patients all underwent a CRS including bilateral salpingo-oophorectomy, hysterectomy, peritoneal staging, omentectomy, pelvic and para-aortic lymphadenectomies and removal of all visible disease. The macroscopic appearance of the omentum, including the presence of disease visible at the time of surgery in different anatomical locations (infra-colic/gastro-colic ligament, PGA), was described. Patients with macroscopic involvement of the PGA were excluded from the study.

**Figure 1.**
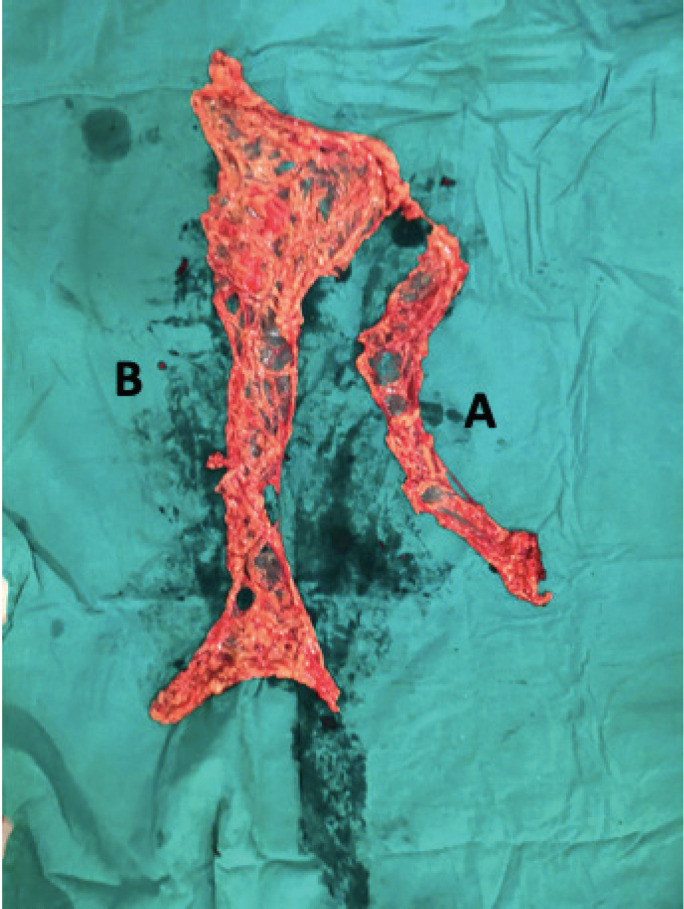
Total omentectomy specimen: (A) peri-gastric arcade specimen (PGA); (B) omentectomy specimen excluding PGA.

**Figure 2.**
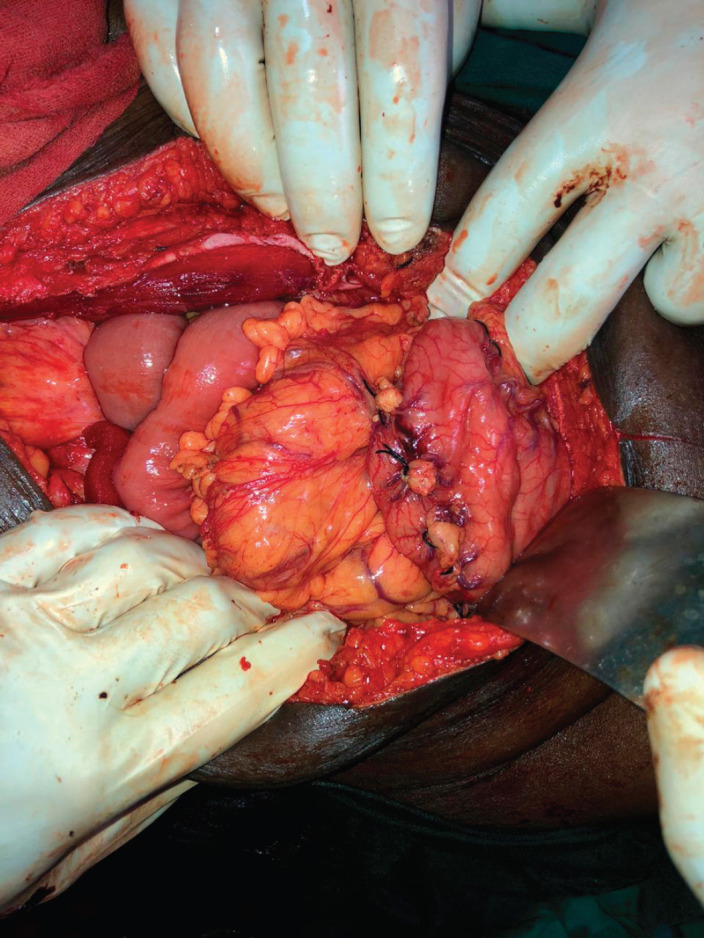
Branches of the peri-gastric arcade near the stomach wall being secured with ligatures.

**Group 1:** Total infra-gastric omentectomy including the vascular PGA (after ligating the right and left gastro-epiploic vessels) of the infra-gastric omental area was performed. This area was excised from the greater omentum specimen and sent separately for histopathological analysis after appropriately labelling the specimen (Figure 1). Ligatures were used to secure the branches of the arcade close to the stomach wall instead of energy sources (Figure 2).

**Group 2:** Total infra-gastric omentectomy excluding the vascular arcade was performed.

Pathological analysis: The peri-gastric area (taken from group 1) was isolated and comprehensively processed for pathological examination. Microscopic involvement of the PGA was tabulated, analysed and conclusions drawn.

Intra-operative factors including duration of omentectomy; stomach, transverse colon or splenic injury and primary haemorrhage were noted. Post-operative course of recovery of group 1 was compared with group 2 and analysed. The parameters used were as follows: number of days for removal of nasogastric tube (NG tube), which was retained till the contents were less than 100 mL; number of days for bowel movements to be established – documented as stated by the patient of having passed flatus; number of days to tolerating oral soft diet; number of days of stay in Intensive Care Unit (ICU); number of days of hospital stay and complications which were classified according to Clavien-Dindo (CD) classification [12].

### Statistical analysis

Data were entered into Microsoft excel data sheet and was analysed using Stata 16 version software. Categorical data were represented in the form of frequencies and proportions. chi-square test was used as a test of significance for qualitative data. Continuous data were represented as mean and SD. Multiple linear regression was used as a test of significance to identify the mean difference between two quantitative variables and qualitative variables, respectively.

*p* value (Probability that the result is true) of <0.05 was considered as statistically significant after assuming all the rules of statistical tests.

Statistical software: MS Excel, StataCorp. 2019. Stata Statistical Software: Release 16. College Station, TX: StataCorp LLC.

## Results

There were a total of 64 patients included in the study, 33 were in group 1 who underwent omentectomy with PGA removal and 31 were in group 2 who had omentectomy with preservation of the PGA. Mean age of patients in group 1 was 50.55 ± 1.95 years, and those in group 2 was 51.35 ± 1.44 years. All the patients, including group 1 and 2, were high-grade serous carcinomas histologically. All the patients in group 1 were in stage IIIC at presentation, while two of the patients in group 2 were in stage IVB at presentation, both of whom underwent NACT followed by cytoreduction. All the patients had preoperative CECT scan with calculation of Meyer’s score [13] which aided in decision regarding primary or interval CRS as described in methodology. Approximately 40% of patients in group 1 and 42% of patients in group 2 had primary CRS, rest had interval CRS (Table 1). All the patients had an assessment of tumour burden with PCI and had total abdominal hysterectomy with bilateral salpingo-oophorectomy followed by recording of completeness of cytoreduction score [14]. The other procedures done included omentectomy (with and without PGA removal – group 1 and 2, respectively), appendicectomy, peritonectomy (pelvic and/or abdominal) and lymphadenectomy (pelvic and/or para-aortic). Complete cytoreduction (CC0) was achieved in 78.8% of group 1 and 74.2% of group 2 (Table 1). There was no statistically significant difference noted between any of these preoperative and intraoperative factors (Table 2). Grossly the mobile part of the omentum (excluding PGA) was involved in 54.5% of group 1 and 61.3% of group 2. Microscopically this part of the omentum was positive for metastasis in 51.5% of group 1 and 54.8% of group 2. These factors also were not significantly different between both groups (Table 1).

Among the 33 group 1 patients, 12 were positive for metastasis in PGA which amounted to 36.4%. Considering the factors associated with microscopic metastasis to PGA, gross involvement of the omentum and microscopic involvement of the mobile part of the omentum excluding the PGA were both found to have significant association with ‘*p*’ values of <0.001. Pre-surgery Meyer’s score and requirement of peritonectomy were the other two factors which had significant association with metastasis to PGA with ‘*p*’ values of 0.004 and 0.024, respectively. Although, the other parameters such as performance of appendicectomy, lymph node dissection, type of surgery or CC score did not show any co-relation as shown in Table 3. Neither did the size of the deposits in the mobile part of the omentum affect the positivity of the PGA. A multiple linear regression was calculated to predict the confounding factors for the microscopic involvement of PGA in patients of group 1. The results showed that none of the positive factors were confounding (Table 4). All factors have independent influence on the outcome. Summarising, grossly abnormal omentum, microscopically positive omentum, pre-surgery Mayer’s score and requirement of peritonectomy can all be considered predictors of metastasis to PGA in the current study.

Looking into the postoperative recovery of the two groups, there was a statistically significant difference between group 1 and group 2 with respect to intraoperative time required for performing omentectomy, number of days to removal of NG tube, post op day of passing flatus, number of days of ICU stay and hospital stay, but no significant difference in number of days to tolerating soft diet (Table 5). There was one patient in group 1 who had primary haemorrhage as a consequence of injury to common iliac vein (unrelated to performance of omentectomy). The injury was detected intra-operatively and repaired but gave away postoperatively as noted by a bloody drain and hence required a re-laparotomy at 36 hours to control the bleed. All the other patients (groups 1 and 2) belonged to CD classification 0 or 1 (minor post-op complications) except for the aforementioned case who fell into class 3b. There was no incidence of injury to stomach, transverse colon or spleen.

## Discussion

The extent of surgery plays a crucial role in deciding survival of ovarian cancer patients. Debulking surgery to the maximal extent is associated with the best overall survival [6]. However, the extent of debulking with CC0can vary from 22% to 98% depending upon various factors [15].

Our study addresses the question of the completeness of cytoreduction and to what extent a surgeon has to go to achieve it especially with respect to omentectomy and removal of PGA. Literature on this particular aspect is sparse. A remarkable study done by Cordeiro Vidal *et al* [10] has served as the benchmark for us.

There is sufficient data from retrospective studies [9, 16, 17] to show that in a grossly normal looking omentum there is micrometastasis in the perigastric area. Hence, the theoretical need to do a prospective study to evaluate this was justified.

Both groups 1 and 2 were well matched with respect to all the preoperative variables as discussed earlier (Tables 1 and 2). Hence, this provided us with a strong platform to draw conclusions about the postoperative parameters.

Twelve out of 33 of our group 1 patients were positive for metastasis in the PGA, amounting to 36.4%. While, the study by Cordeiro Vidal *et al* [10] showed a positivity of 62.5%. This disparity can be explained by the fact that they undertook a more rigorous sectioning and examination of the PGA specimen to look in detail for microscopic metastasis. They also point out that this is not possible in a routine setting. Hence, this called for a more practical approach like in our study where the specimens were subjected to routine pathological sectioning and examination which still resulted in a modest positivity rate of 36.4%.

Yet another merit of our study is that we analysed the factors which seemed to be predictors of metastasis to PGA. These included – grossly abnormal omentum, microscopically abnormal omentum, pre-surgery Meyer’s score and requirement of peritonectomy (Table 3).

Among the patients who had removal of PGA, 54.5% had grossly diseased looking omentum, of which only 6 did not have any microscopic PGA metastases. But all patients who were positive for microscopic deposits in the PGA had diseased looking omentum. Hence, we can extrapolate these findings to say that, two evident intra operative findings – gross involvement of the mobile part of the omentum and/or peritoneum, can foresee a high chance of involvement of PGA. Hence, its removal in these two settings would yield maximum benefits. Notably, in the study by Cordeiro Vidal *et al* [10], 83% of patients with gross disease in the omentum had PGA metastasis.

The number of days in the ICU can be considered as a good marker for immediate postoperative outcomes. As most of these patients are middle aged or elderly, as a routine protocol in our department (both units included), they are shifted to the ICU for monitoring and stabilisation. Haemodynamic stability and non-bloody drain output are considered as criteria to shift the patient to ward. In our study, the group 2 had an average ICU stay of 1.48 days and group 1 had an average stay of 1.6 days (Table 5). The slightly longer operating time (7 more minutes) required for omentectomy with more extensive resection can explain this finding of group 1 patients being in the ICU for a slightly longer duration. When we compare this finding with other studies, they correlate well. In the Cordeiro Vidal *et al* [10] study, the average duration of ICU stay was 1.5 days.

The average hospital stay in our study was 7.61 days in the group 1 and 7.21 days in the group 2 (Table 5). This was found to be significant, but only by a small margin of 0.4 days. The study of Cordeiro Vidal *et al* [10] had an average hospital stay of around 13.2 days. All our patients had CD classification of 0 or 1, indicating minimal postoperative morbidity in both the groups. However, one of our patients in group 1 had to be re-explored for primary haemorrhage (CD-IIIb). In comparison, the Cordeiro Vidal *et al* [10] study had a CD grade III complication in seven patients, these glaring differences between the two studies can possibly be explained by more extensive upper abdominal procedures (resection of spleen and pancreas) in the French study.

Post procedurally, all our patients had a NG tube *in situ*, which was removed only when the drainage was less than 100 mL in 24 hours. Return of bowel functions was indicated by patient passing flatus. We used these two as surrogate markers to assess stomach motility. We found that there were significant differences between the two groups in the day of removal of the NG tube and the day of passing flatus. The patients in group 2 on an average had their NG tubes removed by 2 days and the patients in group 1 had it removed by 2.5 days. Return of bowel function was also faster in group 2 (1.68 versus 2.58 – Table 5). This slightly increased the morbidity of the group with PGA removal, but also served as an effective marker for partial devascularisation of the stomach.

Assessment of gastric motility remains controversial, radionuclide and barium studies have proven to be inaccurate [18]. Hence, we used nasogastric drainage as a surrogate marker of gastric motility.

Further discussing our intraoperative and immediate postoperative outcomes, we noticed that there was no significant difference in the time required for patients to start tolerating a soft diet (Table 5). Patients initially are not able to tolerate a soft diet, as a good proportion of them have nausea and vomiting on the first 2 postoperative days. This is correlated with the study by Cordeiro Vidal *et al* [10], 41.6% of their patients suffered from significant nausea and vomiting in the first two postoperative days. Even though there are significant differences in clinical signs of gastric activity (duration of NG tube and flatus passage) between the two groups, the patient related outcome of tolerating soft diet has no significant difference. This measurement of gastric functions clinically is unique to our study.

Another interesting note is on our technique of omentectomy with the use of ligatures to tie the vessels of the PGA. No energy device was used. This was done to minimise the risk of bleeding postoperatively and any electrical/thermal injury to the stomach wall. Energy device was used only for the inferior part of the omentum in group 2 where PGA was spared. This resulted in more surgical time in group 1 patients, but lead to a safer surgery.

There are a few studies which show that traction on the stomach on the greater curvature to ligate these vessels itself can cause seromuscular injury and lead to gastroparesis [19, 20], but this affect was not observed in our study.

The main strengths of our study include its prospective nature, with two groups (with and without removal of PGA) to evaluate and compare the postoperative outcomes, data to which effect is very sparse. However, in oncology, the effectiveness of any new technique or procedure or modification of a standard procedure is determined by the survival outcomes of the patients [21]. Our study has not been designed to answer this question. Survival is a complex issue with heterogeneous factors influencing it [21]. Hence, there is need of a separate study to define and analyse it. Another limitation is the small sample size when individual arms of the study are considered.

As with any surgical procedure in oncology, it is impossible to eliminate all micrometastasis. In AEOC, there is bound to be micrometastasis all over the abdomen, i.e. peritoneum lining the abdominal organs such as spleen, liver and bowel [6]. As ovarian malignancies have multiple pathways of spread [22], it might be difficult to eliminate all micrometastasis without sacrificing vital organs in the abdomen [23]. However, the principle in ovarian cancer surgery is to eliminate as much disease as possible without causing too much morbidity [24, 15]. Keeping this in mind, it seems reasonable to remove the PGA, as it is technically feasible, not extremely time-consuming and causes little morbidity to the patient in the immediate postoperative period and could potentially improve survival which needs addressing in further studies.

## Conclusion

In our study, micrometastasis to vascular PGA was present in a significant number of AEOC cases. The predictors for this involvement include gross and microscopic involvement of the mobile part of the omentum, pre surgery Meyer’s score and requirement of peritonectomy during the CRS implying that higher the peritoneal carcinomatosis, more are the chances of microscopic involvement of the PGA. The second part of the study deals with the complications of this procedure. Considering that, we noted that although there was a statistically significant difference in intra operative time, prolonged recovery time with increased ICU and hospital stay, there was no significant difference in time taken to tolerate soft diet. Neither were there any major complications attributable to the additional procedure. These observations keep removal of PGA quite a viable option.

Hence, we conclude by stating that removal of vascular PGA during omentectomy is a safe procedure with minimal morbidity and good post-operative outcomes especially in cases with significant peritoneal carcinomatosis; provided we are achieving a CC0otherwise. Further studies are required to prove its survival benefit before we can propose this as a standard procedure.

## List of abbreviations

AEOC, Advanced epithelial ovarian cancer; CC0, Complete cytoreduction; CD, Clavien-Dindo classification; CRS, Cytoreductive surgery; EOC, Epithelial ovarian cancers; ICU, Intensive care unit; NG tube, Nasogastric tube; NACT, Neoadjuvant chemotherapy; PCI, Peritoneal cancer index; PGA, Perigastric arcade.

## Conflicts of interest

There are no conflicts of interest or disclosures.

## Funding

There are no financial disclosures.

## Figures and Tables

**Table 1.** Comparison of intraoperative factors between the groups – nominal variables.

		Group 1		Group 2		*P* value
Type of surgery	Primary CRS	13	39.4%	13	41.9%	0.836
	Interval CRS	20	60.6%	18	58.1%	
Appendicectomy	No	18	54.5%	22	71.0%	0.175
	Yes	15	45.5%	9	29.0%	
Peritonectomy	No	14	42.4%	15	48.4%	0.632
	Yes	19	57.6%	16	51.6%	
Lymph node dissection	No	9	27.3%	11	35.5%	0.516
	Pelvic	5	15.2%	4	12.9%	
	Pelvic + PA	17	51.5%	16	51.6%	
	Pelvic + PA + Inguinal	2	6.1%	0	0.0%	
Completeness of cytoreduction score	0	26	78.8%	23	74.2%	0.617
	1	4	12.1%	4	12.9%	
	2	2	6.1%	4	12.9%	
	3	1	3.0%	0	0.0%	
Grossly positive omentum	Normal	15	45.5%	12	38.7%	0.585
	Abnormal	18	54.5%	19	61.3%	
Microscopically positive omentum	Negative	16	48.5%	14	45.2%	0.79
	Positive	17	51.5%	17	54.8%	

**Table 2.** Comparison of preoperative and intraoperative factors between the groups (Grp 1 and Grp 2) – continuous variables.

		Mean	Std. err.	(95% conf. interval)		*p* value
Age	Grp 1	50.55	1.95	46.66	54.43	0.74
	Grp 2	51.35	1.44	48.47	54.24	
CA 125	Grp 1	307.13	93.08	121.13	493.13	0.45
	Grp 2	452.51	172.40	108.00	797.03	
Pre Sx Mayer Score	Grp 1	1.18	0.13	0.91	1.45	0.15
	Grp 2	1.42	0.09	1.24	1.60	
PCI	Grp 1	5.36	0.87	3.62	7.11	0.63
	Grp 2	4.84	0.61	3.62	6.06	
Omental metastasis size	Grp 1	2.26	0.16	1.95	2.57	0.22
	Grp 2	2.00	0.14	1.72	2.28	

**Table 3.** Predictive factors of microscopic metastasis to perigastric arcade in Group 1.

	Microscopic involvement of perigastric arcade			
		Negative	Positive	*p* value
Type of surgery	Primary CRS	9	4	0.59
	Interval CRS	12	8	
Appendicectomy	No	13	5	0.261
	Yes	8	7	
Peritonectomy	No	12	2	**0.024**
	Yes	9	10	
Gross omental involvement	Negative	15	0	**<0.001**
	Positive	6	12	
LND	No	5	4	0.543
	Pelvic	4	1	
	Pelvic + PA	10	7	
	Pelvic + PA + inguinal	2	0	
Completion of cytoreduction score	0	17	9	0.228
	1	3	1	
	2	0	2	
	3	1	0	
Microscopic omental involvement	Negative	16	0	**<0.001**
	Positive	5	12	
Pre Sx Meyer’s score	0	7	0	**0.004**

**Table 4.** Multiple linear regression for predictive factors of microscopic involvement of perigastric arcade in Group 1.

Microscopic involvement of perigastric arcade	Coef.	Std. err.	*p* value	(95% conf. interval)	
Constant	0.0072	0.1242	0.95	−0.2471	0.2616
Gross omentum	0.0209	0.3759	0.96	−0.7491	0.7909
Micro omentum	0.7082	0.3955	0.08	−0.1019	1.5183
Pre Sx Meyer’s score	−0.0281	0.1368	0.84	−0.3083	0.2520
Peritonectomy	0.0233	0.1480	0.88	−0.2798	0.3264

**Table 5.** Comparison of intraoperative and postoperative course between the two groups (Grp 1 and Grp 2).

Variables		Mean	Std. err.	(95% conf. interval)		*p* value
Intra Op time (omentectomy only in min)	Grp 1	17.30	0.43	16.44	18.17	**<0.001**
	Grp 2	10.48	0.27	9.94	11.02	
Post OP NG tube < 100 ml on day	Grp 1	2.58	0.15	2.27	2.88	**<0.01**
	Grp 2	2.00	0.12	1.75	2.25	
Post op day of passing flatus	Grp 1	2.55	0.14	2.27	2.82	**<0.001**
	Grp 2	1.68	0.11	1.46	1.89	
No of days to tolerating soft diet	Grp 1	3.82	0.13	3.57	4.07	0.104
	Grp 2	3.29	0.08	3.12	3.46	
No of days of ICU stay	Grp 1	1.61	0.14	1.21	1.76	**<0.001**
	Grp 2	1.48	0.17	1.28	1.94	
No of days of hospital stay	Grp 1	7.61	0.25	7.11	8.10	**<0.001**
	Grp 2	7.29	0.15	6.99	7.59	
